# Mapping the spatial variability of HIV infection in Sub-Saharan Africa: Effective information for localized HIV prevention and control

**DOI:** 10.1038/s41598-017-09464-y

**Published:** 2017-08-22

**Authors:** Diego F. Cuadros, Jingjing Li, Adam J. Branscum, Adam Akullian, Peng Jia, Elizabeth N. Mziray, Frank Tanser

**Affiliations:** 1 Deparment of Geography and Geographic Information Science, University of Cincinnati, Cincinnati, USA; 2 Health Geography and Disease Modeling Laboratory, University of Cincinnati, Cincinnati, USA; 3 Biostatistics Program, Oregon State University, Corvallis, USA; 4 Institute for Disease Modeling, 3150 139th Ave SE, Bellevue, USA; 5 Department of Earth Observation Science, Faculty of Geo-Information Science and Earth Observation, University of Twente – ITC, Enschede, Netherlands; 6 World Bank, Washington, DC USA; 7 School of Nursing and Public Health, University of KwaZulu-Natal, Durban, South Africa; 8 Africa Health Research Institute, University of KwaZulu-Natal, Durban, South Africa

## Abstract

Under the premise that in a resource-constrained environment such as Sub-Saharan Africa it is not possible to do everything, to everyone, everywhere, detailed geographical knowledge about the HIV epidemic becomes essential to tailor programmatic responses to specific local needs. However, the design and evaluation of national HIV programs often rely on aggregated national level data. Against this background, here we proposed a model to produce high-resolution maps of intranational estimates of HIV prevalence in Kenya, Malawi, Mozambique and Tanzania based on spatial variables. The HIV prevalence maps generated highlight the stark spatial disparities in the epidemic within a country, and localize areas where both the burden and drivers of the HIV epidemic are concentrated. Under an era focused on optimal allocation of evidence-based interventions for populations at greatest risk in areas of greatest HIV burden, as proposed by the Joint United Nations Programme on HIV/AIDS (UNAIDS) and the United States President’s Emergency Plan for AIDS Relief (PEPFAR), such maps provide essential information that strategically targets geographic areas and populations where resources can achieve the greatest impact.

## Introduction

Mounting evidence suggests a large geographical variation in the sub-Saharan Africa (SSA) human immunodeficiency virus (HIV) epidemic^1–4^. HIV transmission has been shown to be concentrated across clustered micro-epidemics of different scales within a country^2, 4^. This evidence has been aligned with the Joint United Nations Programme on HIV/AIDS (UNAIDS) concept “*know your epidemic, know your response*” for the identification of populations at higher risk of the infection^5^. Identifying areas where the burden of HIV infection is concentrated might play a key role in identifying populations at higher risk of the infection, and knowledge of high and low risk areas is required for successful surveillance programs and optimal resource allocation^6–10^. Moreover, micro-level data on HIV spatial distribution patterns could help to prevent new HIV infections and scale up treatment by prioritizing the allocation of resources to high burden areas and aligning service delivery modalities to the needs of the population.

Other programs such as The United States President’s Emergency Plan for AIDS Relief (PEPFAR) have also gradually shifted their strategies toward optimizing resource allocation by including geographically relevant data as a way of increasing program impact and efficiency^11^. To achieve epidemic control UNAIDS is promoting the 90–90–90 strategy which states that by 2020, 90% of all people living with HIV know their HIV status, 90% of all people with diagnosed HIV infection receive sustained antiretroviral therapy, and 90% of all people receiving antiretroviral therapy have viral suppression. To be able to achieve the 90-90-90 goals it is essential to be able to identify the geographic locations that have the highest HIV burden.

Previous research has assessed the benefits of focusing resources and control interventions using a spatially-targeted allocation strategy^12^. Results support this strategy as being the most efficient for optimal allocation of resources within a country, in contrast to a homogeneous distribution of resources^12–14^. Despite the significance of geographical location in understanding the dynamics of the HIV epidemic and in optimizing resource allocation for its prevention and control, the spatial heterogeneity of HIV in SSA is still not completely understood. Most measures of HIV occurrence are frequently available only for large geographical administrative units. These large-scale (national or regional) measures can obscure localized aspects of the HIV transmission process. A major conclusion from a UNAIDS meeting in 2013 was that generating subnational estimates, particularly high resolution maps of HIV prevalence, would support the urgent need for countries to describe their epidemic at localized levels^15^. The HIV Modelling Consortium was commissioned to address this technical challenge (http://www.hivmodelling.org/projects/ methods-sub-national-estimates-hiv-prevalence), and, in collaboration with modeling groups, UNAIDS representatives, and members of national program teams, evaluated models and methods to achieve the goal of generating detailed subnational geographic estimates of HIV prevalence^16^.

The aim of this article is to use one of the models from the HIV Modelling Consortium to produce high-resolution maps of intranational estimates of HIV prevalence in SSA based on spatial variables.

## Methods

### Data sources

The main source of data for our study was the Demographic and Health Survey (DHS)^17^. We focused our evaluation to countries located at the eastern part of SSA, where the prevalence of HIV is high and there is substantial spatial variability of HIV prevalence within countries^18^. Moreover, countries were included for analysis based on the availability of DHS data from HIV serological biomarker surveys and the corresponding geographic coordinates (latitude and longitude) of sampled locations. For each country, we extracted information from the most recent DHS where HIV data were collected. Data from four countries with generalized HIV epidemic were included, namely Kenya (2008–2009)^19^, Malawi (2010)^20^, Mozambique (2009)^21^, and Tanzania (2011–2012)^22^.

**Figure 1:**
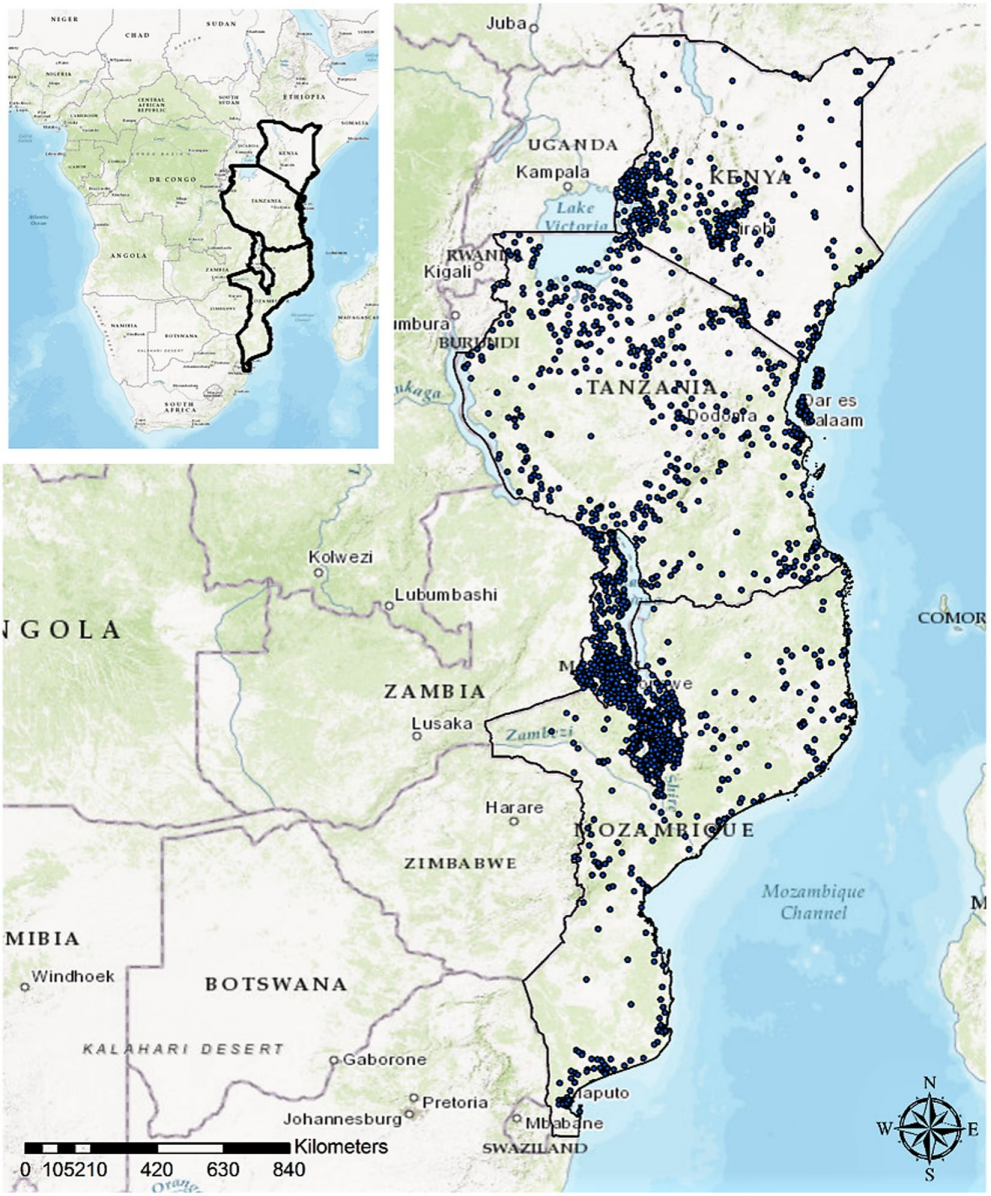
The overall study area (top right panel) and Demographic and Health Survey (DHS) sample locations (blue dots) for each country included in the study. Maps were created using ArcGIS® software by Esri version 10.3^42^ (http://www.esri.com/).

Subjects were enrolled in DHS surveys via a two-stage sampling procedure to select households. In Kenya, 392 DHS sample locations were selected, 832 were selected in Malawi, 269 in Mozambique, and 568 in Tanzania. The global positioning system was used to identify and record the geographical coordinates of each DHS sample location (Fig. 1). Men and women in the selected households were eligible for the study. The study population used in our data analysis consisted of 6,906 individuals in Kenya (3,641 women and 3,265 men), 13,930 individuals in Malawi (7,091 women and 6,839 men), 13,781 individuals in Mozambique (7,895 women and 5,886 men), and 17,745 individuals in Tanzania (9,756 women and 7,989 men).

Anonymous HIV testing was performed with the informed consent of all sampled individuals. HIV serostatus was determined by testing with the enzyme-linked immunosorbent assay (ELISA) Vironostika Uniform 2 Ag/AB. All samples that tested positive and a random sample of 10% of samples that tested negative were retested with a second ELISA, the Enzygnost® HIV Integral II assay (Siemens). Positive samples on both tests were classified as HIV positive. If the first and second tests were discordant, the two ELISAs were repeated; if the results remained discordant, a confirmatory test, the HIV 2.2 western blot (DiaSorin), was administered. Further details related to the DHS methodology, study design, and data can be found elsewhere^22–24^.

### Spatial variable selection

To assess socioeconomic, demographic, and biological variables that may be associated with the spatial distribution of HIV and that have been previously associated with the risk of HIV infection^25–30^, we conducted preliminary bivariate analyses of nine variables extracted from the DHS of each country and other sources. Variables extracted from each DHS included condom use during the last sexual contact, male circumcision, number of lifetime sexual partners, highest educational level, wealth index, and ever been tested for HIV. These variables were evaluated as percentages (calculated using the data collected at each DHS sample location). Specifically, for each sampled location, condom use refers to the percent of individuals who used a condom during the last time having sex, male circumcision refers to the percent of circumcised males, number of lifetime sexual partners is the percent of individuals with less than three lifetime sexual partners, and level of education is the percent of individuals with secondary or higher education. Wealth index is an ordinal variable that describes standard of living as determined by material possessions. The DHS calculated the living standard of a household based on key assets such as television and bicycles, materials used for housing construction, and types of water access and sanitation facilities. The resulting asset scores were used to define wealth quintiles: poorest, poorer, middle, richer, richest. This index was used to calculate a poverty variable as the percent of poorest and poorer people.

Other variables included in the analysis were the normalized difference vegetation index (NDVI), a proxy variable for degree of urbanization that also serves as a measure of potential risk of coinfection with parasitic diseases, which has been shown to increase HIV transmission efficiency in SSA^26, 31, 32^. Distance to main roads and population density were additional variables included in our analysis. All variables were quantified at each DHS sample location. For example, there were 568 DHS sample locations in Tanzania, and the distance from each sample location to the closest main road was calculated (Figure S4). NDVI was obtained from the National Aeronautics and Space Administration (NASA) Earth Observatory Group^33^. The distance to main roads was derived from a road networks dataset sourced from a geographic information system (DIVA-GIS)^34^. Population density was collected from NASA’s Socioeconomic Data and Applications Center^35^.

Data from each country were analyzed by first exploring the randomness of the geographical distribution (spatial structure) of each variable using Moran’s Index for spatial autocorrelation. We then assessed the association between each variable and HIV prevalence by performing bivariate logistic regression analysis. Multivariable logistic regression models were used to examine the associations between selected variables and HIV prevalence in each country using SAS® version 9.3^36^. The dependent variable, *y*_*i*_, was the number of HIV seropositive individuals at sample location *i*. The model posited *y*_*i*_ ~ binomial (*N*_*i*_, *p*_*i*_), where *N*_*i*_ is the total number of individuals sampled and *p*_*i*_ is the HIV prevalence at sample location *i*. The HIV prevalence was related to predictor variables using the multivariable regression model

1 $${logit}({p}_{i})={\beta }_{0}+{\beta }_{1}{X}_{1i}+{\beta }_{2}{X}_{2i}+{\beta }_{3}{X}_{3i}+\ldots +{\beta }_{k}{X}_{ki},$$

where *X*_*ji*_ denotes the value of predictor variable *j* (e.g., percent of circumcised males) at location *i* and *β*_*j*_ is the corresponding regression coefficient.

Variable selection was based on two conditions, namely the bivariate logistic regression coefficient must have p-value < 0.1 and the Moran Index test for spatial autocorrelation must have p-value < 0.05. Population density was included in the final model for each country.

### Mapping Predictor Variables and HIV prevalence

Kriging has been widely used in spatial mapping^37–40^. We used the method of ordinary kriging to predict the values of variables at unmeasured locations by estimating a variogram of weighted averages of the data^41^. Continuous surface maps for each variable are presented in Supplementary Figures 1–4. An HIV prevalence map for each country was then generated by substituting values from all continuous surface maps into the country-specific multivariable logistic regression model using Map Algebra and the inverse logistic equation

2 $$HIV\,Prevalence=\frac{{e}^{{\beta }_{0}+{\beta }_{1}{X}_{1}+{\beta }_{2}{X}_{2}+\cdots +{\beta }_{k}{X}_{k}}}{1+{e}^{{\beta }_{0}+{\beta }_{1}{X}_{1}+{\beta }_{2}{X}_{2}+\cdots +{\beta }_{k}{X}_{k}}}$$

This method was used to generate a map of HIV prevalence in raster format with 5 kilometer grid resolution for each country, utilizing ArcGIS® software version 10.3^42^.

### Model validation

The multivariable logistic regression models used to generate HIV prevalence maps were validated by splitting the data frame into a training data set for fitting models and a testing data set for evaluating model performance using the root mean squared error (RMSE). Training sets contained a simple random sample of 90% of the data; the remaining 10% served as a testing set to evaluate the out-of-sample prediction accuracy of each model. This procedure was repeated 10 times and the average value was used as the final RMSE. Each RMSE was calculated as the square root of the average (across *N* omitted locations) squared difference between the observed HIV prevalence at location *i* (*O*_*i*_) and the estimated prevalence (*E*_*i*_) obtained from equation (1):

3 $$RMSE=\sqrt{\frac{{\sum }_{i=1}^{N}{({E}_{i}-{O}_{i})}^{2}}{N}}$$

A second method of model validation involved exploring and mapping the spatial structure of all residuals (observed value – predicted value) from the multivariable logistic regression models. Specifically, kriging interpolation was used to generate a continuous surface map of the residuals when Moran’s Index indicated significant spatial autocorrelation of the residuals.

### Data availability statement

The data that support the findings of this study are available from the Demographic and Health Surveys (http://www.measuredhs.com) but restrictions apply to the availability of these data, which were used under license for the current study, and so are not publicly available. Data are however available from the authors upon reasonable request and with permission of Demographic and Health Surveys.

### Ethical approval and informed consent

Procedures and questionnaires for standard Demographic Health Surveys (DHS) have been reviewed and approved by the ICF International Institutional Review Board (IRB). Additionally, country-specific DHS survey protocols are reviewed by the ICF IRB and typically by an IRB in the host country. The ICF International IRB ensures that the survey complies with the U.S. Department of Health and Human Services regulations for the protection of human subjects, while the host country IRB ensures that the survey complies with laws and norms of the nation. In the original primary data collection for each DHS, informed consent was sought from all participants prior to serological testing for HIV (http://dhsprogram.com/What-We-Do/Protecting-the-Privacy-of-DHS-Survey-Respondents.cfm#sthash.Ot3N7n5m.dpuf). We sought and were granted permission to use the core dataset for this analysis by MEASURE DHS.

## Results

### Spatial variable selection

The results from bivariate logistic regression analysis and the corresponding Moran’s Index for each variable are presented in Supplementary Table S1. The variables selected for inclusion in the final model for each country are in Table 1. Based on lack of spatial structure and/or statistical significance, condom use and distance to main roads were excluded from the final model for Kenya; condom use, lifetime number of sexual partners and HIV testing were excluded from the final model for Malawi; condom use, level of education, HIV testing, NDVI and distance to main roads were excluded from the final model in Mozambique; and HIV testing and NDVI were excluded from the final model for Tanzania.

**Table 1:** Variables in the final logistic regression model for each country.

Country	Parameter	Estimate	P value	Moran’s I	P value
Kenya	*Intercept*	−2.66	<0.001	−	−
	*Male circumcision*	−0.015060	<0.001	0.20	<0.001
	*Lifetime number of sexual partners*	−0.009149	0.002	0.13	<0.001
	*Level of education*	−0.007512	0.008	0.84	<0.001
	*Poverty*	−0.007524	<0.001	0.51	<0.001
	*HIV Test*	0.023420	<0.001	0.37	<0.001
	*Population density*	0.000007	0.032	−	−
	*NDVI*	0.004674	0.014	−	−
Malawi	*Intercept*	−2.77	<0.001	−	−
	*Male circumcision*	0.006427	<0.001	0.34	<0.001
	*Level of education*	0.009600	<0.001	0.32	<0.001
	*Poverty*	−0.011990	<0.001	0.90	<0.001
	*Population density*	0.000015	0.17	−	−
	*NDVI*	0.013310	<0.001	−	−
	*Distance to main roads*	−0.030720	0.0019	−	−
Mozambique	*Intercept*	−2.21	<0.001	−	−
	*Male circumcision*	−0.010470	<0.001	0.28	<0.001
	*Lifetime number of sexual partners*	0.008017	<0.001	0.38	<0.001
	*Poverty*	−0.013790	<0.0.001	0.71	<0.001
	*Population density*	0.000031	0.001	−	−
	*NDVI*	0.002914	0.044	−	−
Tanzania	*Intercept*	−2.09	<0.001	−	−
	*Condom use*	0.029040	<0.001	0.66	<0.001
	*Male circumcision*	−0.014930	<0.001	0.56	<0.001
	*Level of education*	−0.009500	0.001	0.42	<0.001
	*Lifetime number of sexual partners*	0.014430	<0.001	0.57	<0.001
	*Poverty*	−0.011210	<0.001	0.70	<0.001
	*Population Density*	−0.000015	0.043	−	−
	*Distance to main roads*	−0.030400	0.0017	−	−

### HIV prevalence mapping

**Figure 2:**
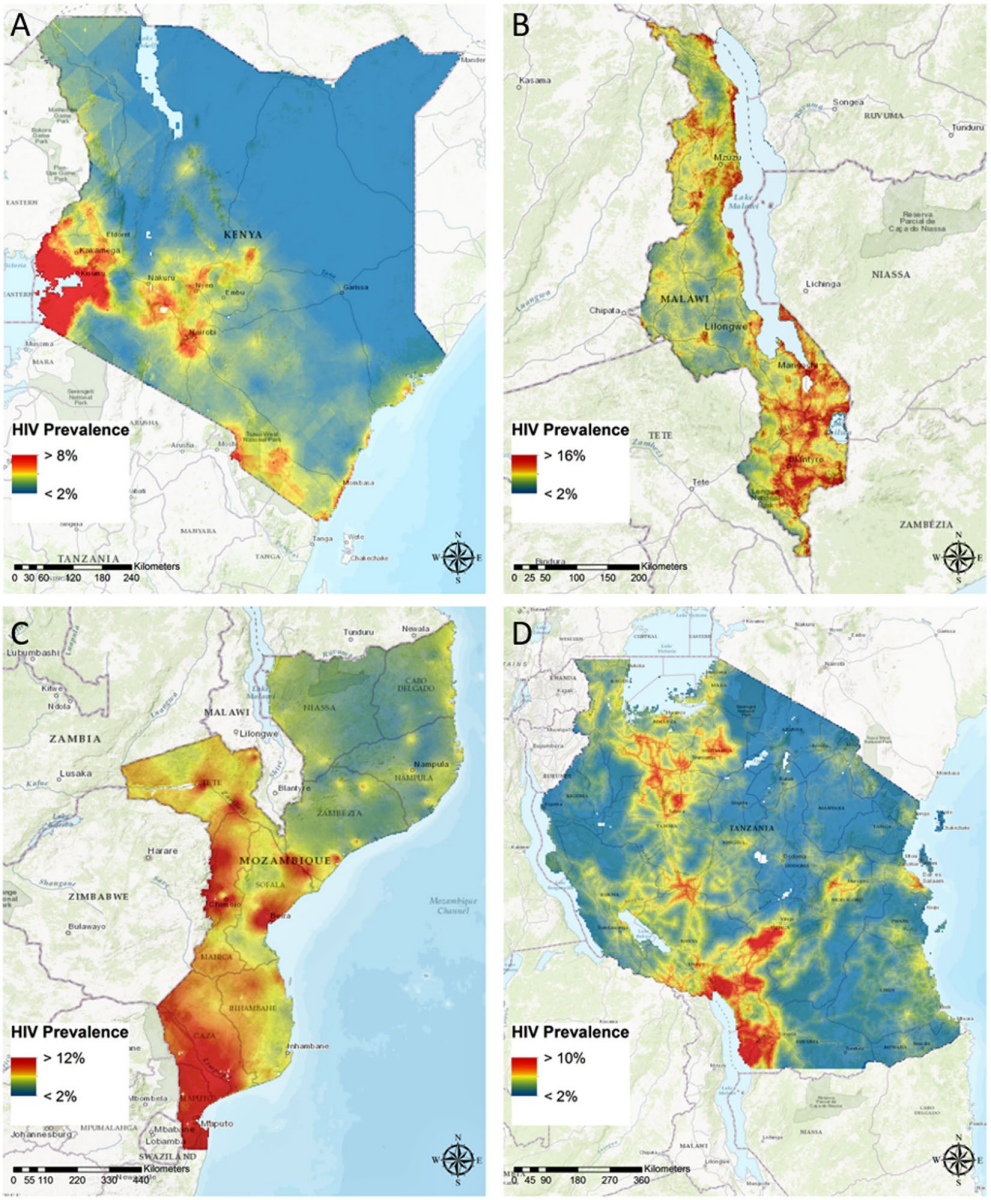
High resolution maps for HIV prevalence in (**A**) Kenya; (**B**) Malawi; (**C**) Mozambique; and (**D**) Tanzania. Maps were created using ArcGIS® software by Esri version 10.3^42^ (http://www.esri.com/).

**Figure 3:**
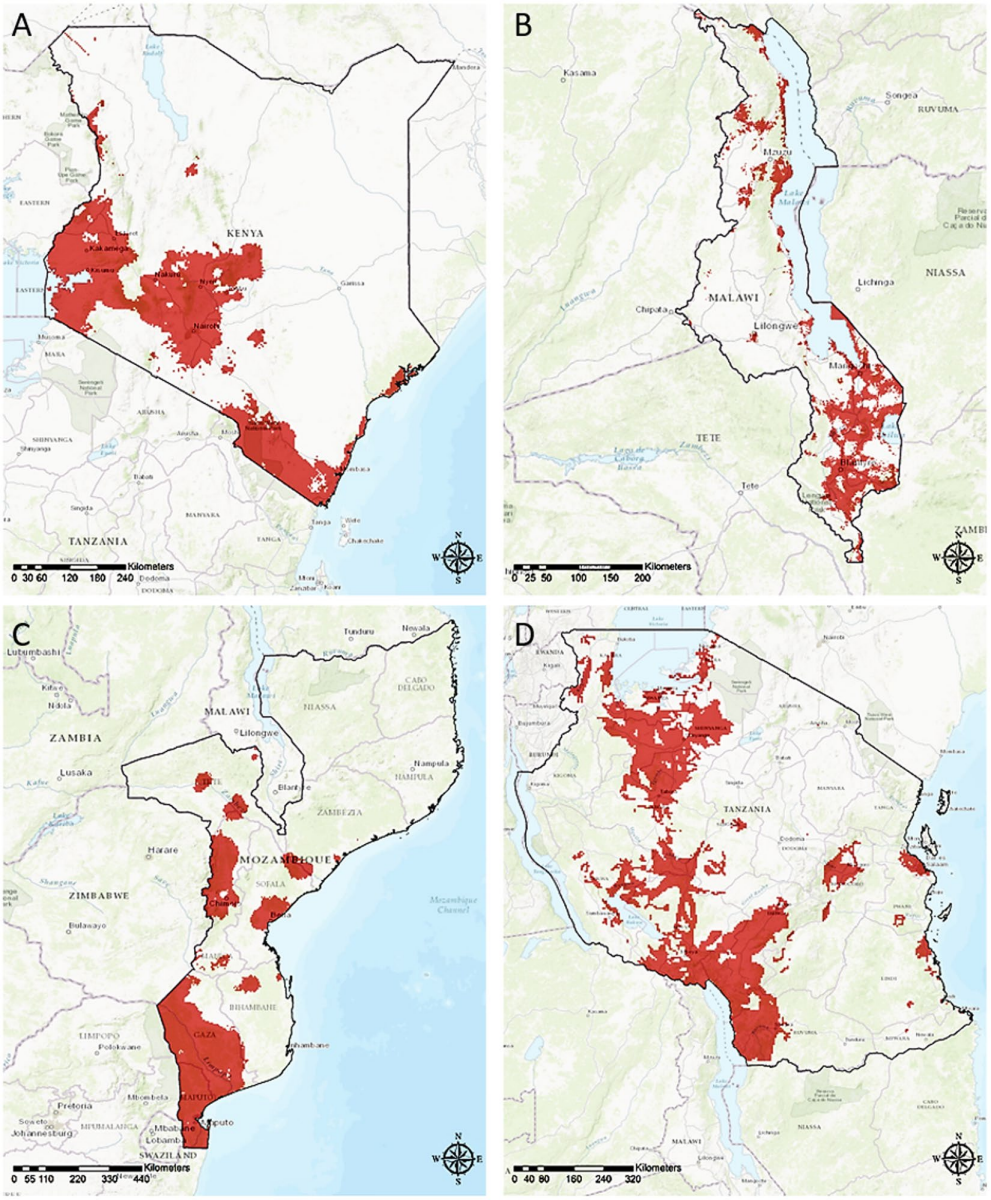
Areas with high HIV prevalence (≥80^th^ percentile) in (**A**) Kenya; (**B**) Malawi; (**C**) Mozambique; and (**D**) Tanzania. Maps were created using ArcGIS® software by Esri version 10.3^42^ (http://www.esri.com/).

HIV prevalence maps generated using the final multivariable logistic regression models for each country are presented in Fig. 2. Figure 3 identifies locations with high HIV prevalence (defined as prevalence ≥ the 80^th^ percentile for the country). Table 2 summarizes the pixel-level HIV prevalence distribution estimations for each country. The estimated pixel-level HIV prevalence distribution in Kenya (Fig. 2A) ranged from 0.5% to 40%, with a median of 2.5%. The vast majority (80%) of locations in Kenya had an estimated HIV prevalence less than 4%, with a median of 2.1%. Figure 3A focuses on the 20% of Kenya with the highest HIV prevalence (median prevalence = 5.2%), where more than 80% of the Kenyan population resides.

**Table 2:** Pixel-level HIV prevalence distribution estimations for each country.

Estimation	Kenya	Malawi	Mozambique	Tanzania
*Entire country*				
Median HIV prevalence	2.5%	8.3%	6.5%	3.2%
Range HIV prevalence	0.5–40.2%	0.6–28.5%	2.5–18.2%	0.5–17.6%
Standard deviation	2.6	2.9	2.7	2.1
*Locations with high HIV prevalence* (*Prevalence* ≥ *the 80th percentile for the country*)				
Median HIV prevalence	5.2%	12.7%	12.4%	6.2%
Range HIV prevalence	4.0–40.2%	11.0–28.5%	11.0–18.2%	5.0–17.6%
Standard deviation	4.3	2.2	1.2	1.7

The pixel-level HIV prevalence in Malawi (Fig. 2B) ranged from 0.6% to 28.5% (median prevalence =8.3%). The HIV prevalence is ≥11% in 20% of Malawi, where more than 19% of the population resides and the median HIV prevalence is 12.7%. In Mozambique (Fig. 2C), the pixel-level HIV prevalence distribution ranged from 2.5% to 18.2% with an 80^th^ percentile of 8%; the median HIV prevalence was 6.5% overall and 12.4% in high-prevalence locations where more than 28% of the population resides (Fig. 3C). The pixel-level HIV prevalence distribution in Tanzania ranged from 0.5% to 17.6% with an 80^th^ percentile of 5%; the median HIV prevalence was 3.2% overall and 6.2% in the high prevalence locations where more than 39% of the population resides (Figs 2D and 3D).

**Figure 4:**
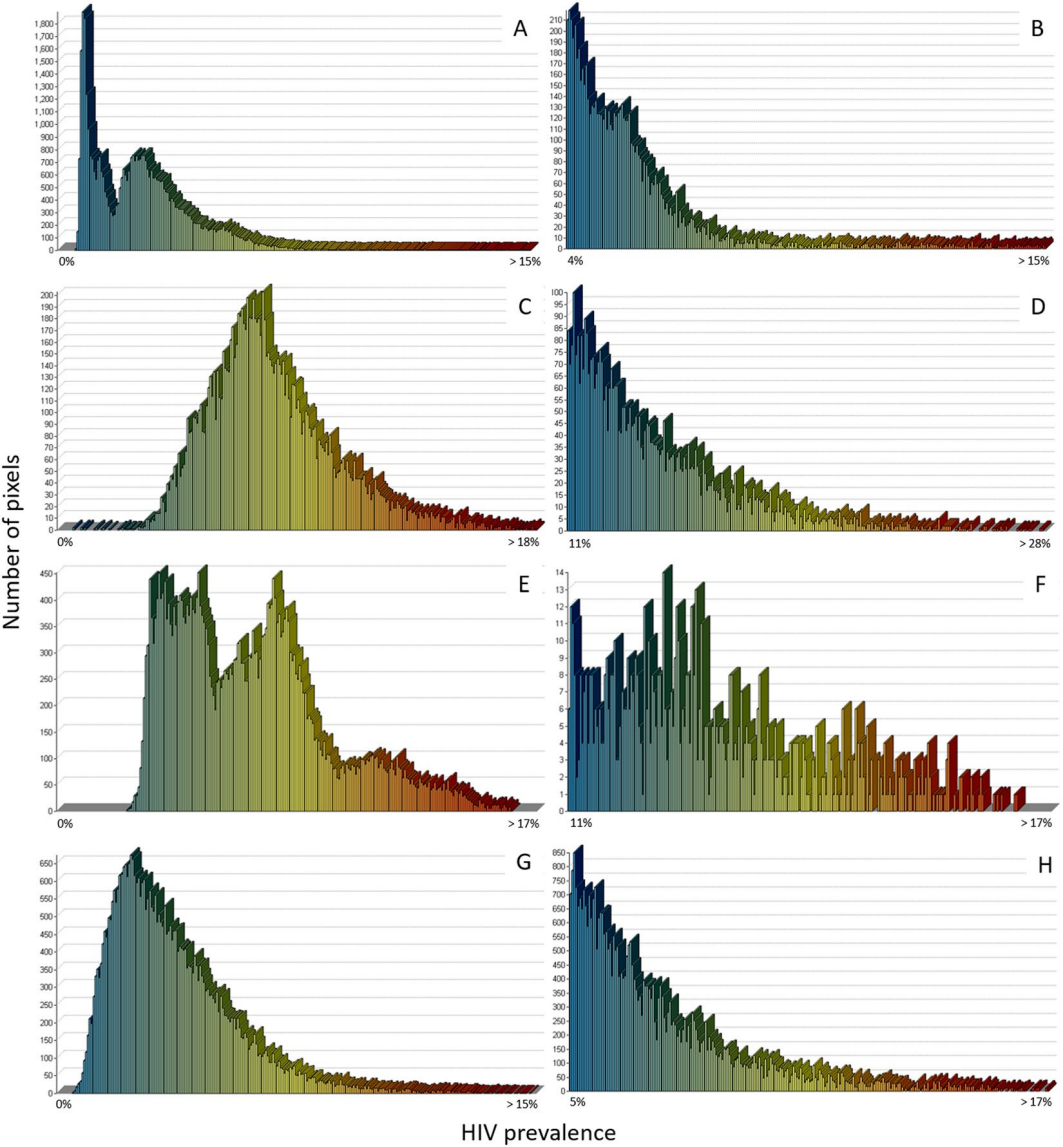
Density distribution of HIV prevalence in Kenya, Malawi, Mozambique and Tanzania for the entire country (left panel; A, C, E, G) and areas with high HIV prevalence (≥80^th^ percentile) in the same country (right panel; B, D, F, H).

The stark geographical variability of HIV prevalence in Kenya, Malawi, Mozambique, and Tanzania is evident from Fig. 4. Kenya and Mozambique had bimodal HIV prevalence distributions (Fig. 4A and E), whereas Malawi and Tanzania had right-skewed distributions and moderate HIV prevalence (Fig. 4C and G). The right-skewed distributions of HIV prevalence in high prevalence areas were similar across countries, and large variation in prevalence was observed in these high burden areas (Fig. 4B,D,F and H).

### Model validation

**Figure 5:**
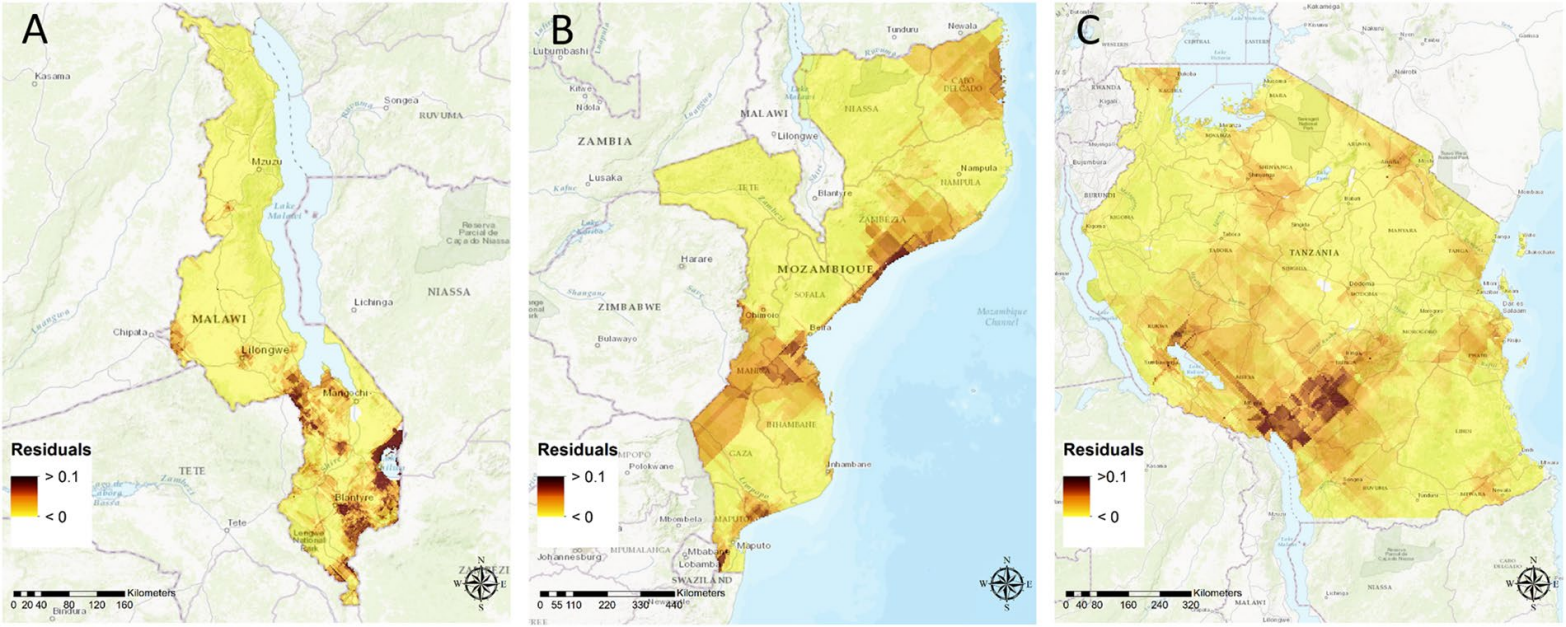
Kriging interpolation of residuals from multivariable logistic regression models for data from (**A**) Malawi; (**B**) Mozambique; and (**C**) Tanzania. Maps were created using ArcGIS® software by Esri version 10.3^42^ (http://www.esri.com/).

Prediction errors indicate that the multivariable logistic regression models were accurate. Specifically, the prediction error was lowest for Tanzania (RMSE = 5.27), followed by Mozambique (RMSE = 6.49), Kenya (RMSE = 8.15), and Malawi (RMSE = 9.09). Moran’s Index identified significant spatial structure in the residuals from the multivariable logistic regression models for Malawi, Mozambique, and Tanzania (*P* < 0.001), but not Kenya (*P* = 0.09). Continuous surface maps of residuals for Malawi, Mozambique and Tanzania suggest that the models could be underestimating the HIV prevalence in areas where prevalence is high (Fig. 5).

## Discussion

The spatial analysis of DHS data on HIV prevalence in Kenya, Malawi, Mozambique, and Tanzania illustrates a rich and complex geographical variation of the HIV epidemic at the subnational level. The high-resolution maps produced from our analysis identified intranational patterns of the spatial distribution of HIV in SSA that would have been missed using macro-level national data. HIV prevalence ranged from 0.5% to 40% across areas in the four SSA countries in this study. In addition, the epidemiological profile of the epidemic in eastern SSA depended on local variation of different spatial variables associated with the risk of HIV. Poverty, NDVI, and distance to main roads were associated with HIV prevalence in some but not all countries included in this study. In contrast to our findings, main highways were shown to be associated with HIV infection in the high-risk subpopulations of female sex workers and their clients in Kenya^43^. Because the data used in our study were obtained from a nationally representative population-based survey, our results pertain to factors associated with HIV prevalence in the general population rather than to specific high-risk subpopulations such as sex workers, which could account for the discrepancy between our results and findings from previous studies.

Male circumcision was the only statistically significant variable associated with HIV prevalence in all four countries. Male circumcision has a distinctive spatial structure that has been previously associated with the geographical distribution of HIV^27^. Furthermore, the spatial distribution of male circumcision along with its spatial association with HIV infection has supported the implementation of interventions such as voluntary medical male circumcision in several countries in SSA^44–47^.

An important finding that underscores the importance of localized data collection and resource allocation is that we did not identify consistent associations between population density and HIV prevalence. In Kenya, the burden of HIV infection appears to be concentrated in areas with high population density (80% of the Kenyan population resides in areas with high HIV prevalence), in contrast to the other three countries where only 19% to 39% of the population resides in high-prevalence areas. Under the premise that in a resource-constrained environment it is not possible to do everything, to everyone, everywhere, this result has important implications for understanding the potential impact of geographically-targeted interventions. Directing prevention and treatment interventions to locations with a relatively small population density but where HIV prevalence is high, such as in Tanzania and Mozambique, could be more cost-effective and have greater impact compared to always directing resources to high population density areas. However, the implications of these findings on programme design and resource allocation is unknown, and future research is needed to establish or refute this conjecture.

The high HIV prevalence areas identified in this study were consistent with the location of geographical clusters or ‘hotspots’ identified using different methods^1, 2, 18^. However, the HIV prevalence maps generated in this study incorporated predictor variable information and they were able to capture greater detail in the spatial structure of those hotspots compared to standard procedures used in spatial clustering analysis.

We found that the spatial heterogeneity of the HIV epidemic in eastern SSA is high even in areas with high HIV prevalence. The distribution of HIV prevalence in high-prevalence areas was skewed and supported very high HIV prevalence values, a result that is in agreement with the spatial variability of HIV observed in hyper-endemic settings such as in rural communities in South Africa^48, 49^. This finding about the highly complex spatial structure of the HIV epidemic in eastern SSA suggests that even in high infection areas, pockets of very high HIV prevalence compared to the national HIV prevalence can emerge. However, there was a rich and complex variation of HIV prevalence among the countries included in our study even in these high HIV burden areas. For example, the median HIV prevalence in Kenya was almost two times higher in areas with high HIV prevalence compared with areas with low HIV prevalence. These high HIV prevalence areas had substantial variation, as indicated by the high standard deviation and a wide range in the HIV prevalence (4.0–40.2%). In contrast, the median HIV prevalence in areas with high HIV prevalence in countries such as Mozambique and Tanzania was also two times higher than the median HIV prevalence in areas with low HIV prevalence within the country, but geographical variation within these high burden areas was much smaller compared to the variation observed in Kenya.

Several study limitations could have affected our results. Given the multiple logistical difficulties in conducting the DHS, some of the variables included in our study could have been affected by inherent biases in the data, such as variability in response rates to HIV testing and under-sampling of mobile individuals and key subpopulations at risk^50, 51^. For instance, some high-risk subpopulations, such as female sex workers, injecting drug users, men who have sex with men, and mobile individuals, could have been missed by the surveys. However, it is not clear to what extent our findings might be affected by under-sampling high-risk populations. Moreover, our results could be affected by patterns of migration and anti-retroviral therapy coverage, information not provided by DHS surveys but that could improve the accuracy of spatial estimation. In addition, the inclusion of data from other sources, such as databases from health facilities or HIV treatment clinics, could improve prevalence estimation accuracy. An additional potential bias is the global positioning system displacement process of the DHS sampling data points, used to preserve their confidentiality^52^. This process could have an impact on the precision of HIV estimations, particularly affecting the location of areas with high HIV burden by several kilometers. Lastly, we included several important variables associated with HIV prevalence that were obtained from reliable sources. However, other variables could have benefited our analysis, such as distance to the nearest health facility and coverage of current programme interventions. We recommend the inclusion of relevant available variables when implementing this methodology at the regional or national level.

Despite these potential limitations, our study highlights the key role that predictor variables might play on the spatial variability of HIV infection. The spatial distribution of these variables provides valuable information for use in generating detailed maps of HIV prevalence distributions. Here, we described straightforward and widely available yet powerful methods for generating intranational estimates of HIV prevalence. These maps illustrate the high spatial variation of the HIV epidemic at subnational levels of eastern SSA. Moreover, these maps enable delineation of areas where the HIV epidemic is concentrated. In an era of limited and declining resources available for HIV prevention and control, it is crucial to focus prevention efforts to key vulnerable populations at the highest risk in areas of greatest HIV burden, as proposed by UNAIDS and PEPFAR^5, 11^. Effective prevention strategies that are currently available include antiretroviral therapy, pre-exposure prophylaxis, voluntary medical male circumcision, and testing and treatment, among others. However, implementation of these prevention strategies throughout the entire general population would present significant challenges, particularly in resource-constrained environments such as in SSA. Therefore, maximizing the impact of prevention programmes on HIV is a priority to reverse the epidemic. Detailed mapping of the spatial structure of the HIV epidemic such as the one described in our study could boost programme efficiency and maximize programme outputs. Such maps provide essential information to strategically target geographic areas to those sub-national units and populations where resources can achieve the greatest impact, and where they are needed the most.

## Electronic supplementary material

Supplementary Materials
