# Supplementary material for: Mapping the spatial variability of HIV infection in Sub-Saharan Africa: Effective information for localized HIV prevention and control
Source: Sci Rep. 2017 Aug 22;7:9093. doi: 10.1038/s41598-017-09464-y (PMC5567213; doi:10.1038/s41598-017-09464-y)
Supplement: Supplementary file 1 — Supplementary Materials [file 41598_2017_9464_MOESM1_ESM.pdf]

Supplementary Materials

Mapping the spatial variability of HIV infection in Sub-Saharan Africa: Effective information for localized HIV prevention and control

Diego F. Cuadros[1, 2*], Jingjing Li[1], Adam J. Branscum[3], Adam Akullian[4], Peng Jia[5], Elizabeth N. Mziray[6], Frank Tanser[7, 8]

[1]*Deparment of Geography and Geographic Information Science, University of Cincinnati, Cincinnati, USA*

[2]*Health Geography and Disease Modeling Laboratory, University of Cincinnati, Cincinnati, USA*

[3]*Biostatistics Program, Oregon State University, Corvallis, USA*

[4]*Institute for Disease Modeling, 3150 139th Ave SE, Bellevue, USA*

[5]*Department of Earth Observation Science, Faculty of Geo-Information Science and Earth Observation, University of Twente – ITC, Enschede, the Netherlands*

[6]*World Bank, Washington, DC, USA*

[7]*School of Nursing and Public Health, University of KwaZulu-Natal, Durban, South Africa*

[8]*Africa Health Research Institute, University of KwaZulu-Natal, Durban, South Africa.*

*To whom correspondence should be addressed. E-mail: diego.cuadros@uc.edu

Supplementary tables

Supplementary Table 1 – Bivariate logistic regression and Moran's Index result for cofactor selection

Country	Parameter	Estimate*	P value	Moran's I	P value
**Kenya**	*Condom use*	0.0300	<0.001	0.81	0.0025
	Male circumcision	-0.0194	< 0.001	0.20	<0.001
	Lifetime sexual partners	-0.0262	0.0024	0.12	<0.001
	Level of Education	0.0007	<0.001	0.84	<0.001
	Poverty	-0.0057	< 0.001	0.51	<0.001
	HIV Test	0.0368	<0.001	0.37	<0.001
	NDVI	0.0099	<0.001	-	-
	Distance to main roads	-0.0309	0.0642	-	-
	Population density	0.0000	0.3000	-	-
**Malawi**	*Condom use*	0.0239	<0.001	0.23	0.2
	Male circumcision	0.0067	<0.001	0.34	<0.001
	Lifetime sexual partners	0.0391	<0.001	0.05	0.9000
	Level of Education	0.0161	<0.001	0.32	<0.001
	Poverty	-0.0143	<0.001	0.91	<0.001
	HIV Test	0.0102	<0.001	0.03	0.9400
	NDVI	0.0151	<0.001	-	-
	Distance to main roads	-0.0620	<0.001	-	-
	Population density	0.0000	<0.001	-	-
**Mozambique**	*Condom use*	0.0189	<0.001	0.27	<0.001
	Male circumcision	-0.0073	<0.001	0.28	<0.001
	Lifetime sexual partners	-0.0024	0.1260	0.38	<0.001
	Level of Education	0.0117	<0.001	0.75	<0.001
	Poverty	-0.0140	<0.001	0.71	<0.001
	HIV Test	0.0204	<0.001	0.95	0.22
	NDVI	-0.0038	0.0042	-	-

	Distance to main roads	-0.0435	<0.001	-	-
	Population density	0.0001	<0.001	-	-
**Tanzania**	*Condom use*	0.0328	<0.001	0.66	<0.001
	Male circumcision	-0.0058	<0.001	0.56	<0.001
	Lifetime sexual partners	0.0161	<0.001	0.57	<0.001
	Level of Education	-0.0042	0.0149	0.42	<0.001
	Poverty	-0.0080	<.001	0.70	<.0001
	HIV Test	0.0264	<.001	0.22	0.09
	NDVI	-0.0033	0.0275	-	-
	Distance to main roads	-0.0285	0.0016	-	-
	Population density	0.0000	0.0742	-	-

*Estimate from the binomial logistic regression

Supplementary figures

Supplementary Figure 1 – Continuous surface maps for the cofactors included in the final model for Kenya. Maps were created using ArcGIS® software by Esri version 10.3* (http://www.esri.com/)

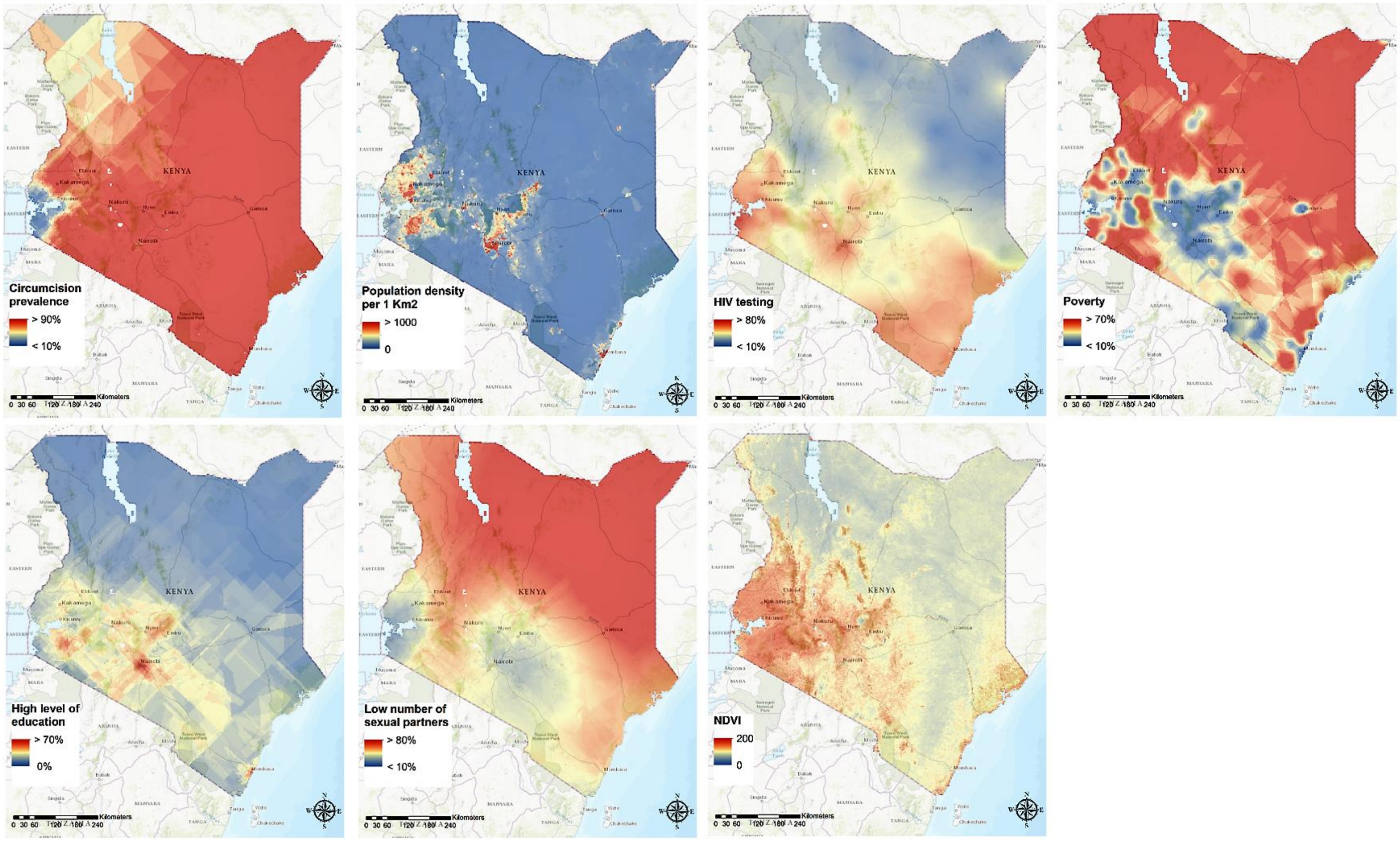

* ESRI. ArcGIS 10.x. Redlands, CA, USA: ESRI.

Supplementary Figure 2 - Continuous surface maps for the cofactors included in the final model for Malawi. Maps were created using ArcGIS® software by Esri version 10.3* (http://www.esri.com/)

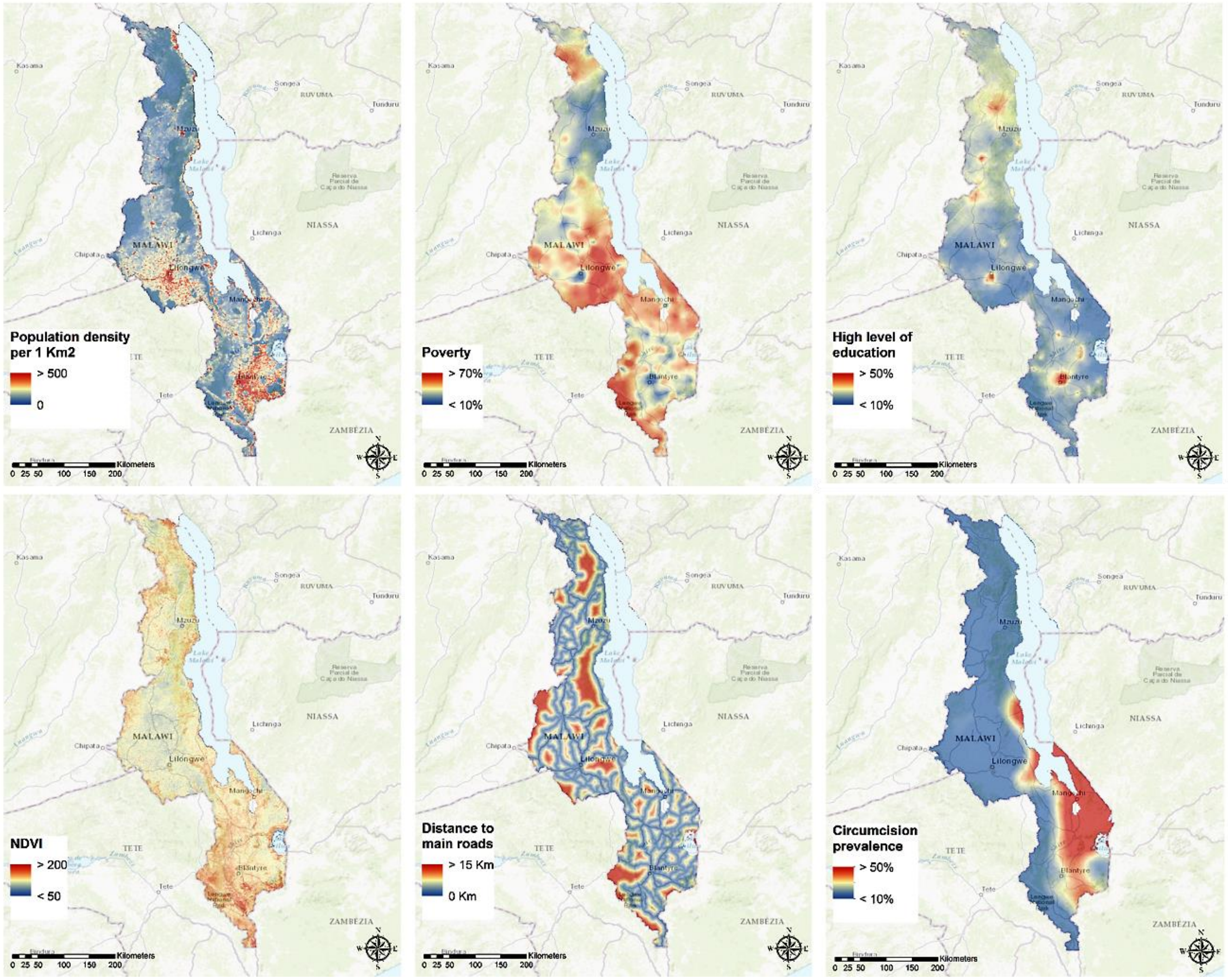

* ESRI. ArcGIS 10.x. Redlands, CA, USA: ESRI.

Supplementary Figure 3 - Continuous surface maps for the cofactors included in the final model for Mozambique. Maps were created using ArcGIS® software by Esri version 10.3* (http://www.esri.com/)

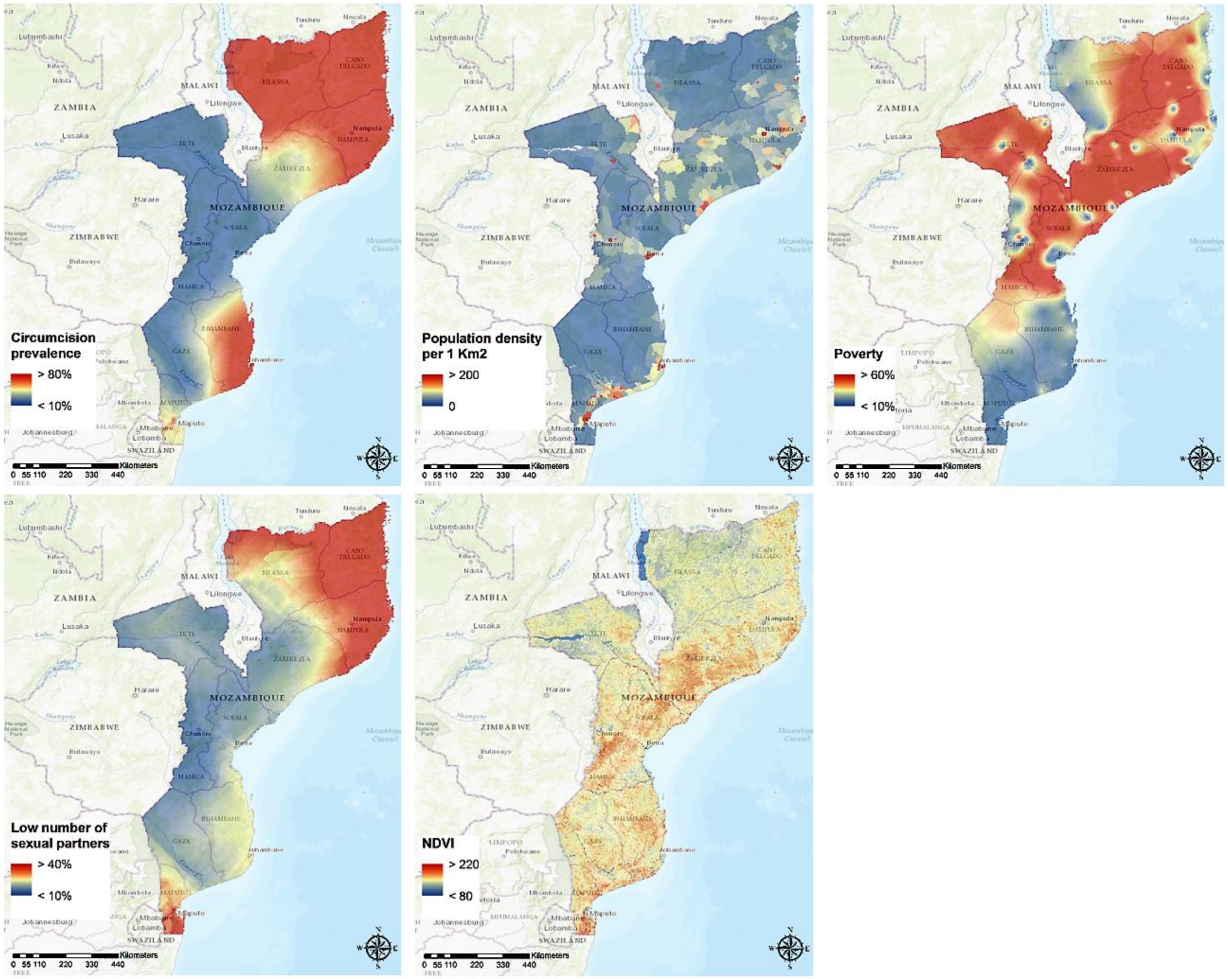

* ESRI. ArcGIS 10.x. Redlands, CA, USA: ESRI.

.

Supplementary Figure 4 - Continuous surface maps for the cofactors included in the final model for Tanzania. Maps were created using ArcGIS® software by Esri version 10.3* (http://www.esri.com/)

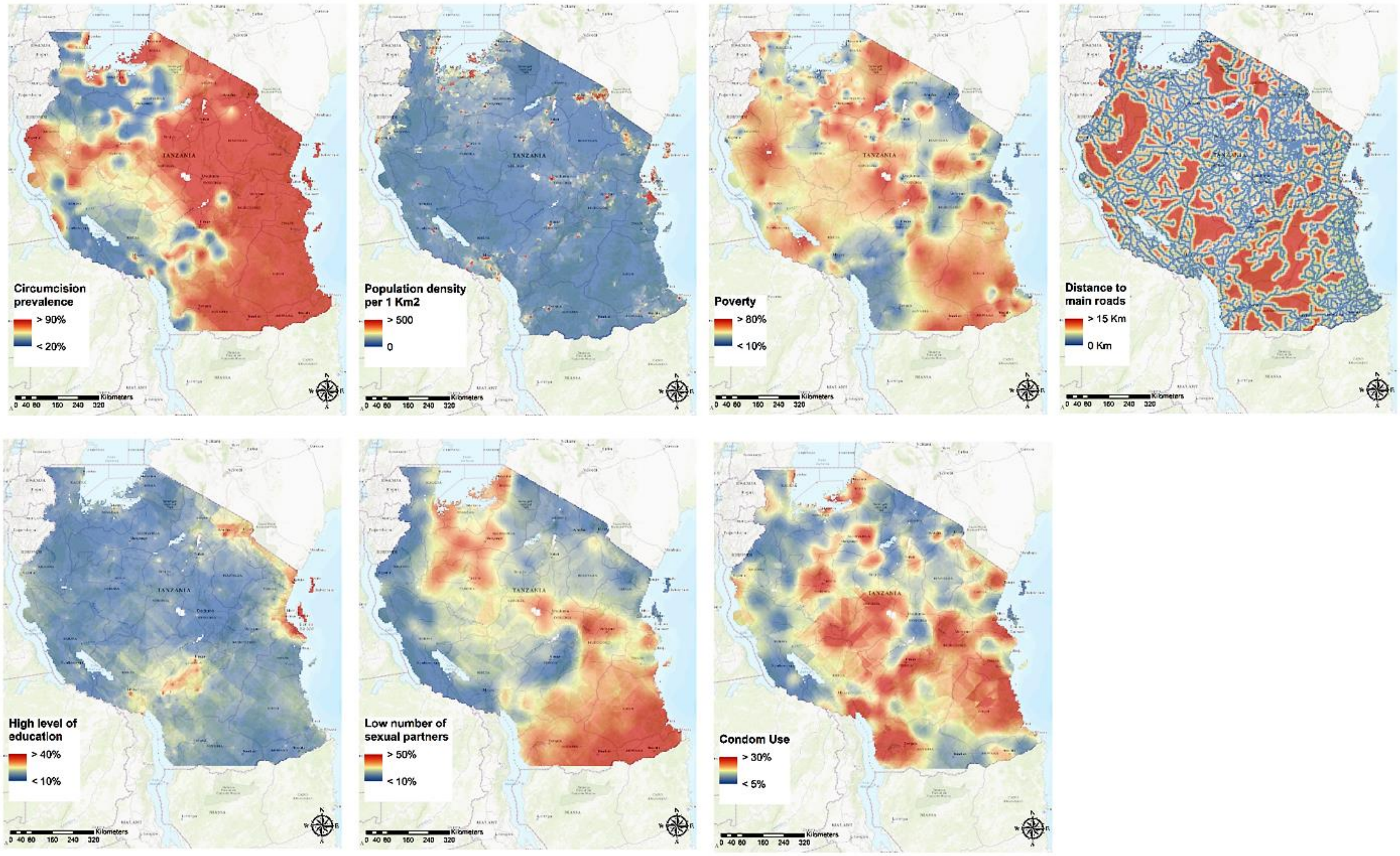

* ESRI. ArcGIS 10.x. Redlands, CA, USA: ESRI.
